# Geschlechtsspezifische Aspekte bei Prädiabetes und Diabetes mellitus – klinische Empfehlungen (Update 2026)

**DOI:** 10.1007/s00508-025-02634-3

**Published:** 2026-04-30

**Authors:** Alexandra Kautzky-Willer, Michael Leutner, Heidemarie Abrahamian, Lisa Frühwald, Fritz Hoppichler, Monika Lechleitner, Jürgen Harreiter

**Affiliations:** 1 Klinische Abteilung für Endokrinologie und Stoffwechsel, Universitätsklinik für Innere Medizin III, Medizinische Universität Wien, Währinger Gürtel 18–20, 1090 Wien, Österreich; 2 Endokrinologische Ordination und Privates Institut für Medizin & NLP, Wien, Österreich; 3 5. Medizinische Abteilung mit Endokrinologie, Rheumatologie und Akutgeriatrie, Klinik Ottakring, Wien, Österreich; 4 Interne Abteilung, Krankenhaus der Barmherzigen Brüder Salzburg, Salzburg, Österreich; 5 Avomed-Arbeitskreis für Vorsorgemedizin und Gesundheitsförderung in Tirol, Innsbruck, Österreich; 6 Abt. für Innere Medizin mit Palliative Care, Landesklinikum Scheibbs, Scheibbs, Niederösterreich Österreich

**Keywords:** Geschlechterdifferenz, Diabetes, Kardiovaskuläre Erkrankungen, Diabeteskomplikationen, Prävention, Sex difference, Diabetes, Cardiovascular diseases, Diabetes-related complications, Prevention

## Abstract

Im klinischen Alltag sind die behandelnden Ärzt:innen mit den unterschiedlichen Bedürfnissen von Männern und Frauen konfrontiert, die in den verschiedenen Lebensabschnitten stark variieren. Geschlechtsspezifische Unterschiede beeinflussen die Pathophysiologie, das Screening und die Diagnose von Diabetes sowie Behandlungsstrategien und die Entwicklung von Komplikationen und die Mortalitätsraten. Veränderungen im Glukose- und Lipidstoffwechsel, die Regulation von Energiehaushalt und Körperfettverteilung sowie Diabetes-assoziierte Begleiterkrankungen werden stark von Steroid- und Sexualhormonen, aber auch von Lebensstil, Umweltfaktoren, Einkommen und psychosozialen Faktoren beeinflusst. Männer weisen im jüngeren Alter und bei niedrigerem BMI ein höheres Risiko für Typ-2-Diabetes (T2DM) auf als Frauen, die wiederum bei Diagnose einen höheren BMI und öfter Hypertonie aufweisen und von einem starken Anstieg im Risiko für kardiometabolische Erkrankungen nach der Menopause betroffen sind. Frauen dürften durch Diabetes auch etwas mehr Lebensjahre verlieren als Männer, wobei die höhere Mortalität hauptsächlich auf vaskuläre Komplikationen zurückgeführt werden kann. Bei Männern mit Diabetes scheint dafür der Mortalitätsanstieg durch Krebs gewichtiger als bei Frauen zu sein. Beide Geschlechter profitieren von Präventionsprogrammen mit einer Risikoreduktion für T2DM. Frauen weisen öfter eine gestörte Glukosetoleranz, Männer hingegen erhöhte Nüchternblutzuckerspiegel auf. Höhere Androgenspiegel sowie ein früherer GDM sind bei Frauen wichtige Risikofaktoren, eine erektile Dysfunktion bzw. niedrige Testosteronwerte bei Männern. Frauen erreichen in der Therapie weniger oft die Zielwerte für HbA_1c_, LDL-Cholesterin oder Blutdruck. In der medikamentösen Behandlung sollen geschlechtsspezifische Unterschiede in der Wirkung, Pharmakokinetik und in den Nebenwirkungen mehr Beachtung finden und individuelle Zielwerte bei Männern und Frauen erreicht werden.

## Abstract (en)

In the clinical routine treating physicians are confronted with the different needs of women and men, which change during their lifetime. Gender-specific differences influence the pathophysiology, screening, diagnostic and treatment strategies of diabetes as well as the development of complications and mortality rates. Alterations in glucose and lipid metabolism, regulation of energy balance and body fat distribution as well as diabetes-associated comorbidities, are greatly influenced by steroidal and sex hormones as well as by lifestyle, environmental factors, income and psychosocial factors. Males appear to have a greater risk for type 2 diabetes (T2DM) at a younger age and at a lower body mass index (BMI) compared to women but women show a higher BMI and more often hypertension at diagnosis and are affected by a dramatic increase in the risk for cardiometabolic diseases after the menopause. The estimated future years of life lost owing to diabetes is somewhat higher in women than men, whereby the higher mortality is mainly attributable to vascular complications. The increased mortality due to cancer in men with diabetes seems to bear more weight than by women. Both sexes benefit from prevention programs with a reduction in the risk for T2DM. Men predominantly feature increased fasting blood glucose levels, women often show impaired glucose tolerance. A history of gestational diabetes or increased androgen levels in women and the presence of erectile dysfunction or decreased testosterone levels in men are important sex-specific diabetes risk factors. Many studies showed that women with diabetes reach their target values for glycated hemoglobin (HbA1c), blood pressure and low-density lipoprotein (LDL)-cholesterol less often than men. Sex differences in the effects, pharmacokinetics and side effects of pharmacological treatment should be taken more into consideration with better achievement of individual target levels in men and women.

## Grundsatz-Statement

Das Geschlecht beeinflusst das Gesundheitsbewusstsein und -verhalten in unterschiedlicher Weise. Neben den biologischen (genetisch und hormonell bedingten) geschlechtsspezifischen Unterschieden sind auch jene als Folge des Einflusses von Gesellschaft, Kultur, Geschlechterrollen und psychosozialen Faktoren zu bewerten und in der Kommunikation, bei der Prävention, der Diagnose und Therapie des Diabetes zu berücksichtigen [1, 2].

## Epidemiologie

In Österreich lag die Lebenserwartung bei Frauen 2023 bei 84,2 Jahren und bei Männern bei 79,4 Jahren [3]. Eine schwedische Studie zeigte keine signifikanten Unterschiede bei Mortalität und Lebenserwartung bei Männern und Frauen mit Typ-1-Diabetes im Vergleich zur südschwedischen Allgemeinbevölkerung [4].

In einer dänischen Studie führte die Entstehung von Typ-1-Diabetes vor dem 10. Lebensjahr bei Frauen zu einem Verlust von 17,7 Lebensjahren (95 %-KI 14,5–20,4) und bei Männern zu einem Verlust von 14,2 Lebensjahren (12,1–18,2) [5].

Die Diabetesprävalenz von 20- bis 79-jährigen Frauen lag 2024 bei geschätzt 10,9 %, was geringfügig niedriger ist als jene der Männer mit 11,3 %. Weltweit haben etwa 9,8 Mio. mehr Männer Diabetes als Frauen [6]. In der österreichischen populationsbasierten Kohortenstudie LEAD war die Prävalenz von Prädiabetes bei Männern 23,6 % verglichen mit Frauen mit 17,1 % [7]. Diabetes wurde bei 7,3 % der Männer und 3,7 % der Frauen diagnostiziert. Mithilfe eines epidemiologischen Diabetes-Progressionsmodells wurde anhand nationaler Gesundheitsdaten von 2012 bis 2016 ein Anstieg der Diabetesprävalenz von 11,7 % auf 12,3 % bei den Frauen und von 14,3 auf 15,1 % bei den Männern über 50 Jahren ermittelt [8]. Die Inzidenzraten nahmen aber bei beiden Geschlechtern über den Zeitraum leicht ab (max. −0,5 %), wenn alle Menschen mit Diabetes, welche ihre Medikation intermittierend pausiert hatten, berücksichtigt wurden. Dabei fanden sich Unterschiede nach Region, Altersgruppe und Geschlecht. Diabetes ist bei Frauen einer der stärksten Risikofaktoren für Herz-Kreislauf-Erkrankungen, die insgesamt 37,1 % der Todesursachen der Frauen und 32,3 % der Männer in Österreich 2023 ausmachten [9, 10]. In einer gepoolten Analyse prospektiver Studien war die Mortalitätsrate bei Männern mit Diabetes krebsbedingt und bei Frauen mit Diabetes vaskulär bedingt besonders stark erhöht im Vergleich zu Gruppen ohne Diabetes gleichen Geschlechts [11]. Ein niedriger Sozialstatus und schlechte Bildung sind mit einem höheren Risiko für Diabetes verbunden. Auswertungen aus der Gesundheitsbefragung in Österreich 2007 ergaben außerdem, dass der inverse Zusammenhang zwischen Bildungsgrad und Auftreten von Übergewicht und Diabetes bei Frauen stärker ist als bei Männern [12].

## Klassifikation und Diagnose

Die Prävalenz von Typ-1- und Typ-2-Diabetes ist bei beiden Geschlechtern annähernd gleich mit einem leichten männlichen Überhang. Lediglich vom Kindesalter bis zur Pubertät scheinen mehr Mädchen als Buben von Typ-2-Diabetes (T2DM) betroffen. In einer rezenten amerikanischen Untersuchung waren Mädchen bei Diagnose auch jünger, und sie wiesen einen milderen Phänotyp auf als die Buben [13]. Dies könnte durch die stärkere Insulinresistenz der Mädchen in der Pubertät bedingt sein sowie durch die Zunahme von Adipositas. In Österreich ist T2DM im Kindesalter selten, allerdings wurde 2021 ein signifikanter Anstieg in der Inzidenz auf 0,5 Fälle per 100.000 Personenjahre festgestellt, wahrscheinlich durch die gleichzeitige Zunahme an Adipositas im Vorschulalter [14, 15]. Interessanterweise wurde in einer anderen Untersuchung auch eine höhere T2DM-Prävalenz bei Mädchen beobachtet, die zugleich mit einer höheren Prävalenz von NAFLD assoziiert war [16]. Im Erwachsenenalter wird bei Männern häufiger und früher und bei niedrigerem Body Mass Index (BMI) als bei Frauen ein Diabetes diagnostiziert [1, 2]. Bezüglich des Stadiums „Prädiabetes“ liegt bei Frauen häufiger das Stadium der gestörten Glukosetoleranz vor, während bei Männern die erhöhte Nüchternglukose überwiegt [1, 17, 18]. Aufgrund dessen ist die Durchführung eines oralen Glukosetoleranztests (oGTTs) bei der Diagnostik von Glukosestoffwechselstörungen v. a. bei Frauen nach wie vor wichtig. Bei Frauen nach Gestationsdiabetes (GDM) zeigten Studien, dass sogar bei einem Großteil eine Glukosetoleranzstörung nur anhand erhöhter 2‑h-Blutzuckerwerte im oralen Glukosetoleranztest erkannt wurde [19]. Zur höheren Rate an gestörter Glukosetoleranz (IGT) von Frauen könnten deren geringere Körpergröße und fettfreie Masse sowie eine verlängerte Darmglukoseaufnahme beitragen [20]. Eine ausgeprägtere Insulinsekretionsstörung in der frühen Phase sowie eine gesteigerte Glukoseausschüttung in der Leber kombiniert mit einer geringeren Insulinsensitivität tragen zu bei Männern häufiger vorkommenden erhöhten Nüchternglukosewerten bei [1]. Prämenopausale Frauen weisen bei vergleichbaren Nüchternglukosewerten im Mittel um 1–1,6 mmol/mol (ca. 0,1–0,15 % HbA_1c_) niedrigere HbA_1c_-Werte auf als gleichaltrige Männer. Dadurch könnten bis zu 17 % der Frauen unter 50 Jahren in Bezug auf eine Diabetesdiagnose unerkannt bleiben. Eine geschlechtsspezifische Anpassung der HbA_1c_-Diagnoseschwelle bei Frauen < 50 Jahren sollte dringend geprüft werden [21, 22].

## Metabolisches Syndrom

Adipositas betrifft in Österreich mehr Buben als Mädchen und Frauen besonders häufig im höheren Alter, während das Vollbild des metabolischen Syndroms je nach Definition bei beiden Geschlechtern unterschiedlich häufig beschrieben wird [15, 23]. Während die IDF-Kriterien annähernd gleich viele Männer und Frauen mit einem metabolischen Syndrom klassifizieren, sind durch die NCEP-ATP III oder WHO-Kriterien mehr Männer als Frauen betroffen [1]. Bei allen Definitionen sind dabei die geschlechtsspezifischen Grenzwerte für HDL-Cholesterin und den Bauchumfang bzw. die „Waist-to-Hip-Ratio“ zu beachten. Unabhängig vom BMI ist ein Bauchumfang über 102 cm bei Männern und über 88 cm bei Frauen mit einer Zunahme des Mortalitätsrisikos um ungefähr 30 % bei den beiden Geschlechtern verbunden [24]. Besonders bei Frauen ist der Bauchumfang ein besserer Prädiktor für Diabetes als der BMI [2]. Eine rezente Studie untersuchte geschlechtsspezifische Unterschiede bei intrahepatischem (HCL) und intramyokardialem Fett (MYCL) sowie der Herzfunktion in Abhängigkeit von BMI und Glukosestoffwechsel. Frauen zeigten bei zunehmendem BMI und zunehmender Verschlechterung des Glukosestoffwechsels stärkere Anstiege von HCL und MYCL, während Männer eine ausgeprägtere Einschränkung der Herzfunktion aufwiesen. Die Ergebnisse deuten darauf hin, dass geschlechtsspezifische Fettverteilungsmuster mit einem erhöhten kardiovaskulären Risiko bei Frauen mit Diabetesprogression assoziiert sein könnten [25].

## Sexualität

Die Prävalenz sexueller Dysfunktion bei Frauen und Männern mit Diabetes ist relativ hoch und weist je nach verwendetem diagnostischem Instrument eine erhebliche Streubreite auf. Die erektile Dysfunktion (ED) ist mit einer Prävalenz von 35–75 % bei Männern mit T2DM die häufigste Form der Sexualfunktionsstörung. Eine kürzlich veröffentlichte Übersichtsarbeit zeigte eine gepoolte Prävalenz von erektiler Dysfunktion bei Männern mit Diabetes von 65,8 % (95 %-KI: 58,3–73,3) [26]. Bei Frauen sind sexuelle Funktionsstörungen seltener untersucht, jedoch werden Beeinträchtigungen der Lubrikation, des sexuellen Verlangens und der Orgasmusfähigkeit häufig berichtet. Eine rezente Metaanalyse ergab, dass das Risiko für sexuelle Funktionsstörungen bei prämenopausalen Frauen mit Typ-1-Diabetes (T1DM) im Vergleich zu Frauen ohne Diabetes signifikant erhöht ist (OR = 3,8; 95 %-KI: 1,8–8,0; *p* < 0,001). Die Prävalenz lag in dieser Metaanalyse in den meisten Studien zwischen 30 und 60 % mit einem Median von 36 % [27]. Die Ätiologie von Sexualfunktionsstörungen ist multikausal und betrifft v. a. vaskuläre, neurogene, hormonelle und psychische Komponenten, deren Zusammenwirken für eine intakte Sexualfunktion Voraussetzung ist. Vaskuläre Funktionsstörungen wie endotheliale Dysfunktion bis hin zur manifesten Atherosklerose sind bei Männern als Ursache einer erektilen Dysfunktion (ED) sehr gut dokumentiert. Männer mit Diabetes mellitus haben ein erhöhtes Risiko für ED [1]. Da in der Regel sowohl eine endotheliale Dysfunktion als auch Atherosklerose bei Gefäßen mit kleinem Lumen früher klinisch manifest werden als bei Gefäßen mit größerem Lumen, wie z. B. Koronargefäßen, wird bei Vorliegen einer ED ein umfassendes Screening auf Makroangiopathie, insbesondere auf koronare Herzkrankheit (KHK), empfohlen [28]. Die ED stellt mit hoher Wahrscheinlichkeit einen unabhängigen Marker für das kardiovaskuläre Risiko dar [1, 29]. Auch die diabetische Polyneuropathie kann zu Sexualfunktionsstörungen bei Männern mit Diabetes beitragen, sowohl als Manifestation im peripheren als auch im autonomen Nervensystem. Dabei spielen oxidativer Stress, ein gestörter Sorbitol-Stoffwechsel und ein Mangel an Nervenwachstumsfaktoren eine wichtige Rolle [30]. Als hormonelle Ursache für Sexualfunktionsstörungen steht der Testosteronmangel mit entsprechenden Auswirkungen auf Libido und Erregung beim männlichen Geschlecht an erster Stelle. Viszerale Adipositas im Rahmen von Diabetes und metabolischem Syndrom sowie die Gesamtfettmasse sind wesentliche Faktoren eines Testosteronmangels bei diesen Patienten [1, 29]. Bei Frauen mit Diabetes sind die kausalen pathophysiologischen Zusammenhänge ähnlich wie bei Männern mit Diabetes gelagert: Endotheliale Dysfunktion und Atherosklerose, Störungen der genitalen Innervation und Veränderungen im Hormon- und Neurotransmittermuster sowie eine ED der Klitoris wurden beschrieben. Darüber hinaus sind bei Frauen auch noch mechanische Irritationen der Schleimhäute durch unmittelbare Auswirkungen der Glykämie mit Beeinträchtigungen der Lubrikation zu berücksichtigen [31]. Obwohl die Prävalenz weiblicher sexueller Dysfunktion bei Frauen mit Diabetes erhöht ist, wird diese Störung im Diabetesmanagement zumeist nicht berücksichtigt und dementsprechend nicht diagnostiziert oder behandelt [32]. Verminderte sexuelle Appetenz („hypoactive sexual desire disorder“ [HSDD] oder „female sexual interest/arousal disorder“ – Nomenklatur nicht einheitlich) ist das am häufigsten geäußerte Problem. Sexuelle Dysfunktion als Nebenwirkung von Medikamenten spielt sowohl bei Männern als auch bei Frauen mit Diabetes eine Rolle. Dabei stehen v. a. bestimmte Antidepressiva mit Auswirkungen auf den Prolaktinstoffwechsel und reduzierter Bildung von Sexualhormonen im Fokus des Interesses. Da bei Menschen mit Diabetes bei ca. 18 % eine behandlungsbedürftige Depression vorliegt, bei Frauen häufiger als bei Männern, ist das jeweilige Antidepressivum sorgfältig auszuwählen, um Nebenwirkungen abzuwenden [33]. Antihypertensiva können die sexuelle Funktion beeinflussen, wobei neuere Studien jedoch überwiegend nur geringe Effekte nachweisen. Unter Berücksichtigung unterschiedlicher Auswirkungen der einzelnen Substanzklassen und geschlechtsspezifischer Aspekte ergibt sich folgendes Bild: Für Männer ungünstig sind Thiazid-Diuretika und ältere Betablocker, zumindest neutral sind Kalziumantagonisten und ACE-Hemmer, potenziell günstig sind AT_1_-Rezeptorantagonisten (Sartane) sowie der Betablocker Nebivolol [34, 35]. Für Frauen ist die Datenlage sehr eingeschränkt. In der SPRINT-Studie (Systolic Blood Pressure Intervention Trial) zeigte sich zwar eine hohe Prävalenz sexueller Dysfunktion, jedoch war keine Assoziation mit einer bestimmten Substanzklasse Antihypertensiva nachzuweisen [35, 36]. Als Screeningmethode empfiehlt sich bei Menschen mit Diabetes zumindest 1‑mal jährlich das sorgfältige Erheben einer Sexualanamnese. Für die ergänzende Diagnostik bieten sich validierte Fragebögen wie International Index of Erectile Function-5-Score (IIEF5) für Männer [37] und Female Sexual Function Index (FSFI) für Frauen an [38]. Beide enthalten Fragen zu den wichtigen Dimensionen der Sexualität wie Libido, Erektion, Lubrikation, Orgasmus, Befriedigung und Schmerz. Bei Vorliegen einer Sexualfunktionsstörung sollte ein kompletter Status der Sexualhormone erhoben werden. Eine erweiterte Vorgehensweise zur Abklärung von Störungen der Sexualfunktion ist im „Informationsblatt für Ärzt:Innen – Anamnese und Diagnostik der sexuellen Dysfunktion bei Diabetes“ dargestellt (Tab. 1). Die Therapie der Sexualfunktionsstörung orientiert sich an den Ursachen, jedoch stehen generell Lebensstilinterventionen und Tabakabstinenz im Vordergrund des Therapieansatzes. Bei vaskulärer Ursache empfiehlt sich die kontinuierliche Therapie der Atherosklerose, bei neuropathischer Ursache ist die Blutzuckereinstellung eine wichtige Maßnahme, bei hormonellen Defiziten v. a. bei Männern kann die Hormonsubstitution in Erwägung gezogen werden. Der Einsatz von PDE-5-Hemmern führt bei Männern zu einer Besserung der Erektion durch Vasodilatation mit gesteigertem Blutfluss durch erhöhte Bereitstellung von Stickstoffmonoxid. Bei Frauen wurde therapeutisch ein gering höherer, möglicherweise klinisch bedeutsamer Benefit für Flibanserin im Vergleich zu Placebo beschrieben, jedoch ist zur optimalen Versorgung eine multidisziplinäre Vorgehensweise erforderlich [32, 39]. Bei Verschreibung von Flibanserin muss eine Aufklärung zu möglichen Nebenwirkungen wie schwere Hypotonie, Schwindel, Synkope und Somnolenz v. a. in Zusammenhang mit Alkoholeinnahme erfolgen [32]. Studien zur Wirksamkeit von Flibanserin bei Frauen mit Diabetes liegen bisher nicht vor, weshalb eine Verschreibung vom/von der behandelnden Ärzt:in in Absprache mit der Patientin hinsichtlich des geringen zu erwartenden Effekts und der möglichen schweren Nebenwirkungen und Kosten abzuwägen ist [32]. Andere therapeutische Möglichkeiten zur Verbesserung der weiblichen Sexualfunktionsstörung wie Testosteron oder Bupropion werden „off label“ eingesetzt und zeigen moderate Effekte. Der Einsatz von PDE-5-Hemmern bei Frauen zeigt kontroverse Ergebnisse und wird nur bei SSRI-induzierter Sexualfunktionsstörung empfohlen [30]. Bei psychischer Ursache der Sexualfunktionsstörung kommt psychotherapeutischen Interventionen eine wesentliche Bedeutung zu. Die hohen Prävalenzzahlen für Sexualfunktionsstörungen bei Menschen mit Diabetes sollten medizinische Fachkräfte und Entscheidungsträger in diesem Bereich dazu ermutigen, der sexuellen Funktionsstörung mehr Aufmerksamkeit zu schenken, indem sie in Versorgungsstrukturen und klinische Leitlinien integriert wird.

**Tab. 1:** Informationsblatt für Ärzt:Innen – Anamnese und Diagnostik der sexuellen Dysfunktion bei Diabetes

Anamnese und Diagnostik der sexuellen Dysfunktion bei Diabetes	
Ein offenes, empathisches Gesprächsklima schafft Vertrauen. Viele Menschen sprechen das Thema von sich aus nicht an, wünschen sich aber, dass der/die Ärzt:in es vorsichtig anspricht	
*Anamnese*	
Vorschläge für niedrigschwellige Einstiegsfragen:	„Viele Menschen mit Diabetes berichten im Laufe der Zeit über Veränderungen im Sexualleben. Haben Sie in diesem Bereich Beschwerden oder Fragen?“ „Wie zufrieden sind Sie derzeit mit Ihrem Sexualleben?“ „Gibt es im intimen Bereich Themen, die Sie belasten oder die Sie ansprechen möchten?“
Medizinische Basisdaten:	Diabetesdauer, HbA_1c_, Folge‑/Begleiterkrankungen (kardiovaskulär, Hypertonie, Dyslipidämie, Neuropathie). Medikamente (z. B. Betablocker, Antidepressiva). Lebensstil (Rauchen, Alkohol, Bewegung, Stress, Schlaf)
Sexualanamnese:	Art der Störung – Erektionsstörung – Ejakulationsstörung – Libidoverlust – Orgasmusstörung – Dyspareunie. Beginn, Verlauf, situative Abhängigkeit. Partnerschaftliche Situation, psychosoziale Belastungen
*Diagnostik*	
Klinisch:	FA für Innere Medizin, Endokrinologe, Urologe, Gynäkologe, Neurologe. Bei starker psychosozialer Komponente Psychotherapeut:Innen, Sexualtherapeut:Innen, klinische Psycholog:Innen, FA für Psychiatrie
Labor:	HbA_1c_, Lipidprofil, Kreatinin, eGFR. Hormonstatus: Testosteron, DHEAS, Östradiol, FSH, LH, SHBG, Prolaktin, TSH
Fragebögen:	IIEF (Männer), FSFI (Frauen). Erfassung von Angst, Depression, Stress
*Besonderheiten bei Diabetes*	
Multifaktorielle Genese: vaskulär, neuropathisch, hormonell, psychogen Interdisziplinäre Abklärung und Betreuung empfohlen	

*DHEAS* Dehydroepiandrosteronsulfat*, eGFR* geschätzte glomeruläre Filtrationsrate („estimated glomerular filtration rate“), *FA* Fachärzt:in, *FSFI* Female Sexual Function Index, *FSH* follikelstimulierendes Hormon, *IIEF* International Index of Erectile Function, *LH* luteinisierendes Hormon, *SHBG* sexualhormonbindendes Globulin, *TSH* Thyreotropin

## Lebensstil und Prävention

Lebensstilmaßnahmen können bei beiden Geschlechtern zu einer Reduktion der Hyperglykämie und Risikoreduktion für Diabetes beitragen [29]. In einer Metaanalyse konnte gezeigt werden, dass eine Lebensstilintervention das Diabetesrisiko bei Männern und Frauen nach 3 Jahren gleichermaßen um etwa 40 % senkt [40]. Im Follow-up der Da-Qing-Studie nach 30 Jahren wurde eine signifikante Reduktion der kumulativen Inzidenz von kardiovaskulären Events um 30 % und der allgemeinen Mortalität um 41 % bei Frauen im Lebensstilinterventionsarm im Vergleich zu weiblichen Kontrollen ohne Lebensstilmaßnahmen gesenkt. Bei Männern konnte hinsichtlich kardiovaskulärer Events und der allgemeinen Mortalität eine nicht signifikante Reduktion um 20 % bzw. 15 % festgestellt werden [41], obwohl die Senkung der kumulativen Diabetesinzidenz bei Männern (39 %) und Frauen (38 %) vergleichbar war. Interessanterweise zeigte sich in der Look AHEAD-Studie, dass übergewichtige/adipöse Männer mit T2DM durch Lebensstilmaßnahmen innerhalb eines Zeitraums von 4 Jahren mehr Gewicht verloren haben [42]. In der randomisiert kontrollierten T4DM Studie konnte gezeigt werden, dass eine Testosteronsupplementation mit 1000 mg Testosteron-Undecanoat bei Männern mit niedrigen Testosteronwerten, IGT oder neu diagnostiziertem T2DM und erhöhtem Bauchumfang im Vergleich zu Placebo die T2DM-Inzidenz nach 2 Jahren Behandlung signifikant um 41 % reduzierte [43]. Bei Frauen scheinen bereits Prädiabetes und das metabolische Syndrom stärker als bei Männern mit erhöhten Inflammationsparametern, einer ungünstigeren Veränderung im Gerinnungssystem und höheren Blutdruckwerten einherzugehen [1, 44]. Dies bestätigt sich auch bei manifestem Diabetes und könnte zum besonders stark erhöhten kardiovaskulären Risiko bei Frauen beitragen [45]. Des Weiteren wurde für Frauen bestätigt, dass anamnestisch erhobene reproduktive Faktoren (Parität, Zyklusunregelmäßigkeiten, Präeklampsie, frühe Menopause) sowie insbesondere die Anamnese eines früheren GDM mit dem Ausmaß der aktuellen Stoffwechselstörung eng assoziiert sind [29]. Frauen mit einem früheren GDM konvertierten bei vergleichbarer Studienausgangslage bezüglich Insulinresistenz, Körpergewicht und Glukosetoleranzstatus fast doppelt so häufig zu manifestem Diabetes wie jene ohne GDM in der Anamnese. Durch Lebensstilmaßnahmen und Metformin kann jedoch die Progression zu T2DM signifikant um fast 40 % im Vergleich zur Kontrolle langfristig reduziert werden [46]. Diese Daten unterstützen bei Frauen die Wichtigkeit der gynäkologischen/geburtshilflichen Anamnese und die Notwendigkeit regelmäßiger, engmaschiger Nachuntersuchung nach GDM (s. auch Leitlinie Management der Hyperglykämie in der Schwangerschaft). Bei Frauen ist das PCOS, das etwa 10 % betrifft, als geschlechtsspezifischer Risikofaktor für Prädiabetes und Diabetes relevant [47]. Es ist gekennzeichnet durch erhöhte Androgenspiegel, Zyklusstörungen und/oder polyzystische Ovarien und geht häufig mit Insulinresistenz einher. Eine Abklärung auf Glukosestoffwechselstörungen ist daher indiziert. Demgegenüber stehen Männer mit niedrigem Testosteronspiegel, die ebenso ein erhöhtes Risiko für Diabetes aufweisen und auch in Screeningmaßnahmen einbezogen werden sollten (s. auch Leitlinie Diabetes mellitus – Definition, Klassifikation, Diagnose, Screening und Prävention) [29].

## Krankheitsbewältigung

Psychosoziale Faktoren beeinflussen die Krankheitsbewältigung und Coping-Strategien bei Männern und Frauen unterschiedlich [2, 48]. Frauen beschäftigen sich generell intensiver mit ihrer Erkrankung und sind besser über Diabetes informiert als Männer, leiden aber mehr unter mit der Erkrankung assoziiertem Stress („diabetes distress“) [2]. Zusätzlich spielen bei Frauen emotionale Faktoren und der Bezug zum/zu der behandelnden Ärzt:in eine größere Rolle. Männer wiederum profitieren besonders von strukturierten evidenzbasierten Diabetes-Management-Programmen. Diabetes verschlechtert die gesundheitsbezogene Lebensqualität bei Frauen stärker als bei Männern [49].

## Orale Antidiabetika, GLP-1-Rezeptoragonisten und Insulin

Frauen erreichen unter Therapie weniger häufig die HbA_1c_-Ziele trotz höheren Risikos für Hypoglykämien [50]. Unter einer Kombinationstherapie mit Metformin und Sulfonylharnstoffen wurde eine stärkere HbA_1c_-Senkung bei Männern berichtet [51, 52]. Die Therapieadhärenz scheint bei Frauen unter einer Metformin-Therapie schlechter zu sein, dies dürfte unter anderem mit einem höheren Nebenwirkungsrisiko zusammenhängen [53]. Im Nebenwirkungsprofil einiger Medikamente gibt es geschlechtsspezifische Unterschiede (Tab. 2). So weisen postmenopausale Frauen unter Glitazon-Therapie trotz eines möglicherweise effektiveren blutzuckersenkenden Effekts im Vergleich zu Männern häufiger Knochenbrüche auf [51]. Die Ursache für diesen Geschlechtsdimorphismus ist bisher unklar, der Einsatz dieser Medikamente sollte v. a. bei postmenopausalen Frauen unter Bedacht erfolgen. Die Erhebung des individuellen Osteoporose- und Frakturrisikos (FRAX) und die Einleitung entsprechender therapeutischer Maßnahmen – sofern erforderlich – werden empfohlen (s. Leitlinie – Diabetes und Osteoporose).

**Tab. 2:** Geschlechtsspezifische Unterschiede in der multifaktoriellen medikamentösen Therapie des Typ-2-Diabetes [56, 106]

Substanz	Bemerkungen
Glitazone	Höheres Knochenfrakturrisiko bei postmenopausalen Frauen
SGLT-2-Hemmer	Harnwegsinfekt, Vulvovaginitis und Balanitis und assoziierte genitale Infekte mit größerem Risiko für Frauen Höheres Risiko für Ketoazidose bei Frauen Vergleichbarer Effekt auf kardiovaskuläre Mortalitätsreduktion und Reduktion der Hospitalisierungen wegen Herzinsuffizienz
GLP-1-Rezeptoragonisten	Höhere kardiovaskuläre Risikoreduktion bei Frauen im Vergleich zu Männern, höhere Häufigkeit von GIT-Beschwerden bei Frauen
Insulin	Frauen: Hypoglykämierisiko nach Insulingabe höher, besonders stark erhöht bei schlanken Frauen Männer: bei normalgewichtigen ebenso höheres Hypoglykämierisiko als bei stark übergewichtigen und adipösen Männern
Lipidsenkende Therapie	Atheromvolumenreduktion bei Frauen mit Rosuvastatin und Evolocumab-Therapie höher Lipidsenkender Effekt bei Frauen unter Fenofibrat größer Statine: Häufiger Nebenwirkungen bei älteren Frauen mit niedrigem Körpergewicht
Thrombozytenaggregationshemmer	Primärprävention: Risikoreduktion von kardiovaskulären Ereignissen um 11 % bei Männern, nicht bei Frauen Allgemein in der Primärprävention keine Empfehlung für Patient:innen mit moderatem kardiovaskulärem Risiko oder hohem Alter
β‑Blocker	Erhöhte Blutdruck- und Herzfrequenzreduktion bei Sport betreibenden Frauen
Kalziumkanalblocker	Stärkere Blutdrucksenkung bei Frauen Erhöhte Ödeminzidenz bei Frauen
ACE-Inhibitoren	Erhöhte Reizhusteninzidenz bei Frauen

*SGLT‑2* Sodium-Glucose-Co-Transporter 2, *GIT* Gastrointestinaltrakt, *GLP‑1* „glucagon-like peptide 1“, *ACE* „angiotensin converting enzyme“

Unter Basalinsulintherapieeinsatz wurden besonders bei nur leicht übergewichtigen Frauen häufiger schwere Hypoglykämien beschrieben [50], vermutlich aufgrund höherer Insulindosen bezogen auf das Körpergewicht. Dies trifft aber auch für normalgewichtige und leicht übergewichtige Männer zu. Des Weiteren erreichen Frauen unter einer Basalinsulintherapie seltener als Männer den HbA_1c_-Zielwert von < 7 % [54]. In der Substanzklasse der SGLT-2-Hemmer sind häufiger Ketoazidosen, insbesondere aber Harnwegsinfekte und Genitalinfektionen, v. a. Pilzinfektionen, bei Frauen beschrieben (Risiko für Frauen bereits vor Beginn einer SGLT-2-Hemmertherapie 3‑fach höher als bei Männern, unter Therapie bei beiden Geschlechtern erhöht), v. a. wenn schon früher rezidivierend Infektionen im Urogenitalbereich aufgetreten sind [2]. Bei Patient:innen mit einer neu diagnostizierten Kombination aus T2DM und kardiovaskulärer Erkrankung ist das weibliche Geschlecht mit einer geringeren Einleitung kardioprotektiver Antidiabetika assoziiert – insbesondere bei Vorliegen einer koronaren Herzkrankheit oder Herzinsuffizienz [55]. In allen kardiovaskulären Outcomestudien zu SGLT-2-Hemmern oder GLP-1-Rezeptoragonisten waren insgesamt weniger Frauen eingeschlossen, die auch im Beobachtungsverlauf weniger kardiovaskuläre Ereignisse zeigten. Die kardiovaskuläre Risikoreduktion war bei Frauen mit Diabetes in allen diesen kardiovaskulären Outcomestudien nicht signifikant, obwohl keine Interaktion mit dem Geschlecht gefunden und positive Trends bestätigt wurden [56]. Dies dürfte wohl durch die geringere Anzahl an Studienteilnehmerinnen sowie die generell niedrigere Rate an kardiovaskulären Ereignissen im Beobachtungszeitraum bei den Frauen und der daraus resultierenden niedrigeren statistischen Power bedingt sein. Jedenfalls bestätigte eine gepoolte Analyse von Outcomestudien für beide Substanzklassen eine signifikante Senkung des kardiovaskulären Risikos (primäre kardiovaskuläre Outcomes) bei Männern wie auch bei Frauen [57]. Auch in einer systematischen amerikanischen Übersichtsarbeit erwies sich der positive Effekt auf die kardiovaskuläre Mortalität und Hospitalisierung wegen Herzinsuffizienz bei Männern und Frauen als vergleichbar [58]. In prospektiven kontrollierten Studien reduzierte Empagliflozin das Risiko für kardiovaskuläre Todesfälle und Hospitalisierungen aufgrund einer Herzinsuffizienz gleichermaßen bei Frauen und Männern mit einer HFpEF [59]. Auch unter Dapagliflozin konnten vergleichbare positive Effekte bei Frauen und Männern mit einer Herzinsuffizienz mit mäßiggradig eingeschränkter EF oder HFpEF aufgezeigt werden [60]. Trotzdem wurde gezeigt, dass SGLT-2-Hemmer bei Frauen mit T2DM selbst bei bekannter KHK, Nieren- oder Herzinsuffizienz, ebenso wie generell bei sozial benachteiligten Menschen seltener angewendet wurden [61]. Im Vergleich zu Sulfonylharnstoffen waren sowohl DPP-4-Hemmer, GLP-1-Rezeptoragonisten als auch SGLT-2-Hemmer mit weniger kardiovaskulären Ereignissen (Myokardinfarkt/instabile Angina, Schlaganfall oder Herzinsuffizienz) bei beiden Geschlechtern assoziiert [62], wobei bei Frauen durch GLP-1-Rezeptoragonisten signifikant bessere kardiovaskuläre Effekte erzielt wurden als bei Männern. GLP-1-Rezeptoragonisten zeigen quantitative geschlechtsspezifische Unterschiede in der Wirksamkeit, wobei Frauen generell stärker ansprechen. Trotz wachsender Anwendung bei Frauen fehlen weiterhin ausreichende Studienergebnisse [63]. Es besteht eine mögliche Interaktion mit Östrogenen, und Unterschiede zeigen sich auch in der Wirkung auf Appetit, Stoffwechsel, Stimmung und Reproduktionsfunktion. Auch im SURPASS-Programm mit Tirzepatid (GIP- und GLP-1-Rezeptor-Agonist) konnte bei Erwachsenen mit T2DM dosisabhängig eine bei Frauen stärkere Gewichtsabnahme erzielt werden. Zu den Prädiktoren für eine Gewichtsabnahme > 15 % zählte neben einem jüngeren Alter v. a. das weibliche Geschlecht (OR 2,6).

## Multifaktorielles Risikomanagement

Frauen mit Diabetes erreichen weniger häufig die leitlinienkonformen Therapieziele für HbA_1c_, Blutdruck und/oder Lipide [56, 64]. Das könnte auf Unterschiede in der Rate an Komorbiditäten, an Krankheitssymptomen, in der ärztlichen Einschätzung der Gefährdung der Patient:innen bzw. dem ärztlichen Kommunikations- und Verordnungsmodus, in der Therapieadhärenz oder aber auch in der allgemein höheren Nebenwirkungsrate in der Pharmakotherapie bei Frauen zurückgeführt werden. Allerdings reagieren Frauen auf bestimmte kardiovaskuläre Risikomarker sogar empfindlicher im Risikoanstieg für Komplikationen als Männer. Frauen haben eine höhere Salzsensitivität und reagieren auf Salzzufuhr mit einem deutlicheren Blutdruckanstieg, profitieren aber auch stärker bei Salzrestriktion. Ebenso führen hohe Serumtriglyzeride und niedriges HDL-Cholesterin bei Frauen zu einem höheren Anstieg des kardiovaskulären Risikos. Rauchen ist bei Frauen mit einem um 25 % höheren Risiko für Myokardinfarkt verbunden als bei Männern. Rauchen sollte daher v. a. bei Diabetes unbedingt vermieden werden. Unterschätzte kardiovaskuläre Risikofaktoren sind der GDM, das polyzystische Ovarsyndrom (PCOS) und eine Schwangerschaftshypertonie oder Präeklampsie, die bei Frauen mit Diabetes öfter auftreten, sowie eine frühe Menopause [2, 9]. Junge Frauen mit Zustand nach GDM haben ein 2‑fach höheres Risiko für kardiovaskuläre Events [65]. Bei Frauen steigt das Risiko für Adipositas, Dyslipidämie, Hypertonie und Glukosestoffwechselstörungen nach der Menopause deutlich an, weswegen ein regelmäßiges Monitoring dieser Risikoparameter in dieser Phase besonders wichtig ist [9, 66]. Besorgniserregend ist, dass insbesondere Hochrisikopatientinnen mit KHK eine schlechtere Kontrolle modifizierbarer kardiovaskulärer Risikofaktoren aufweisen und weniger häufig eine intensive lipidsenkende Therapie erhalten als Männer mit KHK [17, 56, 67]. Eine systematische Übersichtsarbeit mit randomisiert kontrollierten kardiovaskulären Outcomestudien bei T2DM zeigte deutlich ein schlechteres kardiovaskuläres Risikomanagement mit höheren systolischen Blutdruckwerten, höherem LDL-Cholesterin und höherem HbA_1c_ bei den Frauen [68]. Frauen erhielten auch weniger häufig Statine, Betablocker oder Acetylsalicylsäure (ASS) [68]. Statine wirken bei Frauen und Männern annähernd gleich [69]. In einer rezenten und bisher größten Metaanalyse konnte für beide Geschlechter ein vergleichbarer Benefit hinsichtlich vaskulärer und nichtvaskulärer Outcomes durch Atorvastatin und Rosuvastatin in der primären und sekundären Prävention beobachtet werden [70]. Die SATURN-Studie zeigte unter Hochdosis–Rosuvastatin-Therapie eine signifikant höhere prozentuale Atheromvolumenreduktion bei Frauen im Vergleich zu Männern [71]. Die LDL-C-Senkung mit PCSK9-Hemmern fiel in einigen Studien bei Frauen signifikant geringer aus bei Männern, war aber dennoch hocheffektiv (ca. 50 % Senkung von LDL-C) [72, 73]. In der CLEAR-Outcome-Studie, in der 48 % Frauen eingeschlossen wurden, zeigte sich in Subanalysen ein vergleichbarer Effekt hinsichtlich LDL-Cholesterinsenkung und MACE-Reduktion mit Bempedoinsäure zwischen Frauen und Männern [74, 75]. Unter einer Fenofibrat-Therapie im Vergleich zu Placebo war der lipidsenkende Effekt bei Frauen mit T2DM ausgeprägter als bei Männern und mit einer – wenn auch nicht signifikant unterschiedlichen – höheren Reduktion kardiovaskulärer Events verbunden [76]. Finerenon reduzierte bei Menschen mit mäßiggradig reduzierter Herzinsuffizienz oder HFpEF bei beiden Geschlechtern gleichermaßen das Risiko für kardiovaskuläre Todesfälle oder Hospitalisierungen aufgrund von Herzinsuffizienz [77]. In einer Post-hoc-Analyse von FIDELITY wurde bei Menschen mit T2DM und CKD, geschlechtsspezifisch gesehen, ein gleichermaßen reduziertes Risiko für nierenspezifische und kardiovaskuläre Outcomes unter Finerenon präsentiert. Bei Hospitalisierungen für Herzinsuffizienz hatten Männer einen geringen Vorteil gegenüber Frauen [78]. Bei den Antihypertensiva ist zu berücksichtigen, dass Frauen für die Auslösung von Arrhythmien durch QT-verlängernde Substanzen empfindlicher sind und bei Betablockern oft niedrigere Dosen als Männer benötigen. ACE-Hemmer scheinen bei Frauen die kardiovaskuläre Mortalität weniger stark zu senken, dafür aber eine Nephropathieentwicklung stärker zu verzögern, während AT-Rezeptor-Antagonisten bei Frauen besser wirksam sein könnten [79]. Eine rezente Post-hoc-Analyse von Geschlechterunterschieden in der Wirksamkeit von AT-Rezeptorblockern auf Nieren- und Herz-Kreislauf-Erkrankungen bei Typ-2-Diabetes und diabetischer Nierenerkrankung bestätigte eine nephroprotektive Wirkung bei Männern und Frauen [80]. Das kardiovaskuläre Ergebnis konnte allerdings nur bei Männern (HR 0,81), aber nicht bei Frauen vermindert werden (Geschlecht × Behandlungsinteraktion HR 1,37).

## Thrombozytenaggregationshemmer

Eine Therapie mit ASS ist bei Frauen mit einer geringeren antithrombotischen Wirkung und zudem auch mit einem höheren Blutungsrisiko assoziiert. ASS reduziert in der Primärprävention bei Frauen im Gegensatz zu Männern nicht das Myokardinfarktrisiko, wohl aber das Risiko für ischämische Insulte [81]. Eine systematische Übersichtsarbeit zeigte bei Männern in der Primärprävention kardiovaskulärer Ereignisse eine signifikante relative Risikoreduktion von kardiovaskulären Ereignissen (MACE) von 11 %, wohingegen bei Frauen der Effekt nicht signifikant war [82]. Viele Studien zeigen, dass Frauen mit kardiovaskulärem Risiko seltener ASS erhalten als Männer, obwohl für Frauen mit Diabetes in der Sekundärprävention ebenso wie für Männer ASS empfohlen wird (75–325 mg/Tag). In der Primärprävention sind die Daten weniger klar. Für Männer und Frauen mit Diabetes kann zwischen 50 und 70 Jahren auf individueller Basis und nach entsprechender Aufklärung und Nutzen-Risiko Abwägung eine Therapie mit ASS (75–162 mg/Tag) überlegt werden [83, 84]. In der ASCEND-Studie konnte zwar eine Reduktion von vaskulären Ereignissen unter ASS 100 mg bei Menschen mit Diabetes mellitus in der Primärprävention festgestellt werden, jedoch war auch die Ereignisrate für Blutungen um 29 % erhöht. Geschlechtsspezifische Unterschiede wurden weder bei Risikoreduktion vaskulärer Ereignisse noch im Blutungsrisiko festgestellt [85]. Aufgrund des erhöhten Blutungsrisiko und des geringen Nettobenefits in rezent publizierten RCTs und Metaanalysen wird in internationalen Leitlinien die Verwendung von ASS in der Primärprävention, v. a. in höherem Alter (> 70 Jahre) und bei moderatem Risiko für kardiovaskuläre Ereignisse (auch bei Diabetes mellitus) nicht empfohlen [86]. Bei Hochrisikopatienten kann ASS in Erwägung gezogen werden.

## Komplikationen und Komorbiditäten

### Makrovaskuläre Komplikationen

Während bei Männern mit und ohne Diabetes mellitus die kardiovaskuläre Mortalität im letzten Jahrzehnt abnahm, bleibt die Rate bei Frauen mit Diabetes unverändert hoch oder steigt sogar tendenziell an [1, 87]. Frauen mit Diabetes mellitus haben v. a. bei Diagnosestellung häufiger eine fortgeschrittene Atherosklerose verglichen mit Männern [51]. Ein höheres Risiko für Tod durch KHK ist bei Frauen mit Diabetes bekannt (Tab. 3). Neuere Studien konnten zeigen, dass diese geschlechtsspezifischen Unterschiede hinsichtlich kardiovaskulärer Mortalität, aber auch der Entwicklung einer Herzinsuffizienz bei Diabetes mellitus weiterhin bestehen und in jüngeren Altersgruppen sogar noch deutlich ausgeprägter sind [88–90]. Daher scheinen auch Forderungen nach intensiveren Bemühungen für Screeningmaßnahmen für Glukosestoffwechselstörungen und anderen kardiovaskulären Risikofaktoren sowie der frühzeitigen Etablierung prophylaktischer Maßnahmen gerechtfertigt [90]. Nach einem Myokardinfarkt haben Frauen eine schlechtere Prognose. Dabei dürfte v. a. der fehlende protektive Effekt der weiblichen Geschlechtshormone eine Schlüsselrolle einnehmen. Die Symptome eines akuten Koronarsyndroms sind bei Frauen oft komplex und untypisch mit stärkerer vegetativer Ausprägung (Müdigkeit, Abgeschlagenheit, Übelkeit, Hals‑, Kiefer- oder Rückenschmerzen etc.) und werden deshalb häufiger fehlinterpretiert; Hypertonie ist besonders bei Frauen mit Diabetes ein wichtiger Risikofaktor für KHK, aber auch für Herzinsuffizienz. Daraus folgt, dass die Blutdruckkontrolle bei Frauen strikt verfolgt werden muss. Eine Metaanalyse stellte bei Frauen und Männern mit T1DM oder T2DM ein höheres Risiko für Herzinsuffizienz im Vergleich zu gesunden Frauen und Männern fest [91]. Im direkten Vergleich zu Männern haben aber Frauen mit T1DM oder T2DM das höhere relative Risiko (T1DM 47 %, T2DM 9 %). Auch das Risiko für eine Hospitalisierung wegen Herzinsuffizienz ist bei Frauen mit T2DM höher [68, 92]. In einer niederländischen Observationsstudie wurde eine 25 % höhere Inzidenzrate für den primären Endpunkt (Mortalität oder Hospitalisierung wegen Herzinsuffizienz) bei Männern mit bekannter chronischer Herzinsuffizienz beobachtet. Komorbiditäten wie Diabetes mellitus, Niereninsuffizienz oder chronische Lungenerkrankungen zeigten jedoch bei Frauen mit bekannter chronischer Herzinsuffizienz stärkere Assoziationen mit dem primären Endpunkt [93]. In einer österreichischen Observationsstudie zeigte sich, dass der NYHA-Score bei Frauen mit T2DM im Gegensatz zu Männern kein unabhängiger Prädiktor für die Herzinsuffizienzdiagnose war [94]. Hingegen war ein bereits gering erhöhter NT-proBNP-Wert bei Frauen mit einer höheren Mortalitätsrate assoziiert: Während sich kein signifikanter Unterschied in der Sterblichkeit bei Männern mit NT-proBNP-Werten zwischen 125 und 400 pg/ml und solchen unter 125 pg/ml zeigte, war bei Frauen die Sterblichkeit in der Gruppe mit NT-proBNP-Werten zwischen 125 und 400 pg/ml bereits signifikant höher als darunter.

**Tab. 3:** Geschlechtsspezifische Unterschiede bei diabetesassoziierten Komplikationen und Komorbiditäten. (Nach [1, 107])

Parameter	Anmerkungen
Komorbiditäten	Frauen: allgemein höhere Belastung
Körperliche und kognitive Einschränkung/geriatrische Aspekte	Frauen: körperliche und kognitive Limitationen, Depressionen und Stürze häufiger
Depression/Angst	Frauen: höhere Depressions- und Angstprävalenz bei Diabetes Männer: stärkerer Zusammenhang zwischen Diabetes und Depression
Schwangerschaftskomplikationen (Frauen mit GDM oder T2DM)	Siehe Leitlinie Gestationsdiabetes und Gravidität bei vorbestehendem Diabetes
Nephropathie	Männer: schnellere Progression Frauen: höheres Risiko einer Proteinurie und Nierenerkrankung
Retinopathie	Männer mit T1DM: bei Manifestation nach dem 15. Lebensjahr stärkeres Risiko für Entwicklung einer proliferativen Retinopathie und Nierenversagen, Männer: höheres Risiko bei T2DM
Sensorische Neuropathie	Männer: höheres Risiko bei T2DM
Diabetischer Fuß	Männer: höheres Risiko für Fußulzerationen, peripher vaskulären Komplikationen und Neuropathie
Koronare Herzkrankheit	Frauen: 40 % höheres relatives Risiko für koronare Herzkrankheit, letale und nicht letale Events
Herzinsuffizienz	Frauen: höheres relatives Risiko
Schlaganfall	Frauen: 27 % höheres relatives Risiko für Schlaganfall, letale und nicht letale Events

*GDM* Gestationsdiabetes, *T1DM* Typ-1-Diabetes, *T2DM* Typ-2-Diabetes

Frauen leiden generell öfter unter einer diastolischen, Männer öfter unter einer systolischen Dysfunktion. Frauen leiden auch wesentlich häufiger an einer Herzinsuffizienz mit erhaltener Ejektionsfraktion (HFpEF) [9, 95]. Empagliflozin konnte auch in dieser Gruppe in der EMPEROR-Preserved-Studie den primären Endpunkt, eine Kombination von kardiovaskulärem Tod und Hospitalisierung aufgrund der Herzinsuffizienz, erreichen. Eine geschlechtsspezifische gepoolte Metaanalyse ergab, dass die Effekte von SGLT-2-Hemmern auf vaskuläre Risiken und Tod, ebenso wie auf unerwünschte Nebenwirkungen zwischen Männern und Frauen vergleichbar war [96]. Eine geschlechtsspezifische Analyse der TOPCAT-Studie (52 % Frauen) zeigte, dass Frauen im Gegensatz zu Männern über das ganze Spektrum der Herzinsuffizienz von einer Spironolacton-Therapie profitieren [97], im amerikanischen Studienarm war die Gesamtmortalität nur bei Frauen signifikant vermindert [98]. In der PARAGON-HF-Studie (52 % Frauen) wurde unter Therapie mit ARNI (Sacubitril Valsartan) in einer präspezifizierten Subgruppenanalyse nur bei Frauen eine signifikante (−27 %) Reduktion des primären Endpunkts (kardiovaskulärer Tod und Hospitalisierung aufgrund der Herzinsuffizienz) erreicht, auch wenn das Ergebnis der verminderten Hospitalisierungsrate zuzuschreiben war [99, 100]. Die Frauen wiesen öfter Adipositas, weniger oft eine koronare Herzkrankheit und Diabetes (40 vs. 46 %) auf und waren stärker symptomatisch. Die nichtinvasive Diagnostik der KHK hat bei Frauen eine besonders niedrige Sensitivität und Spezifität, insbesondere die Ergometrie ist wenig aussagekräftig. Oft liegen auch trotz Beschwerden blande Koronarien im Katheter vor (MINOCA), da v. a. bei jüngeren Frauen mikrovaskuläre funktionelle Störungen oder Spasmen überwiegen [101]. Provokationstests und Stresstests können pathologische Veränderungen aufzeigen.

### Mikrovaskuläre Komplikationen

BMI, Alter und höhere Blutzuckerwerte scheinen bei Männern stärkere Prädiktoren für einen Nierenfunktionsverlust darzustellen. Zu beachten ist des Weiteren, dass Frauen mit Diabetes ein besonders hohes Risiko für Harnwegsinfekte haben, welche konsequent behandelt werden müssen. Bei Retinopathie und Neuropathie sind bisher nur wenige Geschlechtsunterschiede beschrieben (Tab. 3). In der rezent publizierten Maastricht-Studie konnte ein höheres Risiko für Nephropathie, Retinopathie und sensorische Neuropathie bei Männern mit T2DM im Vergleich zu gesunden Männern festgestellt werden. Dies war bei Frauen mit T2DM im Vergleich zu gesunden Frauen nicht der Fall [102].

### Tumoren

Diabetes ist mit einem höheren Krebsrisiko verbunden, wobei Übergewicht eine zusätzliche wichtige Rolle spielt. Frauen mit Diabetes haben ein höheres Risiko für Brustkrebs und ein doppelt so hohes Risiko für Endometriumkarzinome, während bei Männern das Risiko für Prostatakarzinom etwas niedriger ist [103]. Außerdem ist bei beiden Geschlechtern das Risiko für Pankreaskarzinome, Darmkrebs und Leberkrebs deutlich erhöht. Frauen mit Diabetes nehmen seltener an Vorsorgeuntersuchungen (Mammographie) teil [104]. Bei beiden Geschlechtern ist auf die Durchführung der allgemein empfohlenen Screeninguntersuchungen unbedingt zu achten.

### Osteoporose

Diabetes ist mit einem höheren Osteoporose- und Frakturrisiko assoziiert, wobei der Knochenstoffwechsel und die Knochenqualität – selbst bei erhaltener Knochenmasse – ungünstig verändert sind. Männer mit Neuropathie scheinen besonders gefährdet [105]. Männer und Frauen mit Diabetes sollen auf ihr individuelles Osteoporoserisiko untersucht werden.

### Depressionen

Diabetes ist häufig mit depressiven Störungen verbunden, welche bei Frauen doppelt so häufig wie bei Männern diagnostiziert werden, aber bei Männern häufig nicht erkannt werden. Auch Angststörungen und kognitive Einschränkungen werden häufiger bei Frauen mit Diabetes mellitus beobachtet [1]. Es soll deshalb bei beiden Geschlechtern regelmäßig auf das Vorliegen einer Depression geprüft werden (s. Leitlinie psychische Erkrankungen).

## Zusammenfassung

Auch wenn derzeit noch viele Fragen in Bezug auf biologische und psychosoziale geschlechtsspezifische Aspekte in der Entstehung, Prävention und Therapie des Diabetes offen sind und eine wichtige Aufgabe und Herausforderung für zukünftige Forschung darstellen, muss dennoch auf Basis der derzeitigen stetig zunehmenden Erfahrungen und Erkenntnisse bereits eine geschlechtssensible medizinische Betreuung der Patient:innen in der Praxis mit Ziel der Personalisierung der medizinischen Betreuung gewährleistet werden. Insbesondere ist auf eine konsequente leitlinienkonforme Therapie modifizierbarer kardiovaskulärer Risikofaktoren bei beiden Geschlechtern zu achten. Dies sollte auch bereits in jüngeren Altersgruppen konsequent umgesetzt werden.

## Interessenkonflikt

A. Kautzky-Willer hat von folgenden Unternehmen, die teils auch fördernde Mitglieder der ÖDG sind, Forschungsunterstützungen und/oder Honorare erhalten: Amgen, Bayer, Boehringer Ingelheim, Eli Lilly, Merck, Novartis, Novo Nordisk, Roche. M. Leutner hat von folgenden Unternehmen, die auch fördernde Mitglieder der ÖDG sind, Honorare erhalten: Abbott, AstraZeneca, Bayer, Boehringer Ingelheim, Daiichi Sankyo, Novartis, Novo Nordisk. L. Frühwald hat Vortragshonorare und Übernahme von Kongressgebühren erhalten von E. Lilly, Sanofi, Boehringer Ingelheim, Novo Nordisk, Abbott. F. Hoppichler hat von folgenden Unternehmen, die auch fördernde Mitglieder der ÖDG sind, Honorare erhalten. AstraZeneca, Eli Lilly, Novartis, Novo Nordisk, Sanofi-Aventis. J. Harreiter hat von folgenden Unternehmen, die auch fördernde Mitglieder der ÖDG sind, Honorare erhalten: Amarin, Amgen, AstraZeneca, Bayer, Boehringer Ingelheim, Novartis, Novo Nordisk, Ultragenyx. H. Abrahamian und M. Lechleitner geben an, dass kein Interessenkonflikt besteht.
